# Effects of climate on dental mesowear of extant koalas and two broadly distributed kangaroos throughout their geographic range

**DOI:** 10.1371/journal.pone.0201962

**Published:** 2018-08-22

**Authors:** Larisa R. G. DeSantis, Jagger Alexander, Eva M. Biedron, Phyllis S. Johnson, Austin S. Frank, John M. Martin, Lindsay Williams

**Affiliations:** 1 Department of Earth and Environmental Science, Vanderbilt University, Nashville, Tennessee, United States of America; 2 Department of Anthropology, Vanderbilt University, Nashville, Tennessee, United States of America; Max Planck Institute for Evolutionary Anthropology, GERMANY

## Abstract

Dental mesowear analysis can classify the diets of extant herbivores into general categories such as grazers, mixed-feeders, and browsers by using the gross wear patterns found on individual teeth. This wear presumably results from both abrasion (food-on-tooth wear) and attrition (tooth-on-tooth wear) of individual teeth. Mesowear analyses on extinct ungulates have helped generate hypotheses regarding the dietary ecology of mammals across space and time, and recent developments have expanded the use of dental mesowear analysis to herbivorous marsupial taxa including kangaroos, wombats, possums, koalas, and relatives. However, the diet of some of the most ubiquitous kangaroos (e.g., *Macropus giganteus*) along with numerous other species cannot be successfully classified by dental mesowear analysis. Further, it is not well understood whether climate variables (including precipitation, relative humidity, and temperature) are correlated with dental mesowear variables including various measures of shape and relief. Here, we examine the relationship between dental mesowear variables (including traditional methods scoring the sharpest cusp and a new potential assessment of multiple cusps) and climate variables in the grazers/mixed feeders *Macropus giganteus* and *Macropus fuliginosus*, and the obligate browser *Phascolarctos cinereus*. We find that dental mesowear of mandibular teeth is capable of differentiating the dietary habits of koalas and the kangaroo species. Furthermore, both *Macropus giganteus* and *Phascolarctos cinereus* exhibit mesowear correlated with mean minimum temperature, while *Macropus fuliginosus* dental mesowear is unaffected by temperature, despite significant differences in mean minimum and mean maximum temperature across their distribution (and in the specimens examined here). Contrary to expectations that individuals from drier regions would have blunter and lower relief teeth, dental mesowear is unrelated to proxies of relative aridity—including mean annual precipitation and relative humidity. Collectively, dental mesowear in these marsupials is related to feeding behavior with increased wear in cooler regions (in *Macropus giganteus* and *Phascolarctos cinereus*) potentially related to more or different food resources consumed.

## Introduction

The diet and foraging habits of organisms are critical components of their ecology and often determine where they occur. Understanding the diets of fossil organisms can provide information about long-term trends in vegetation structure and organismal responses to climate change. Multiple paleoecological proxy methods, including dental microwear, dental mesowear, and stable isotope analysis, are used to determine the textural properties and isotopic composition of dietary items (e.g., [1–3]). While dental microwear and stable isotope analysis require specialized equipment and a high level of training, dental mesowear is comparatively inexpensive and can be taught to novices in under an hour [4]. Dental mesowear analysis involves examining the cusp shape and relief of teeth to determine the diets of herbivorous mammals (e.g., grazing, mixed feeding, and browsing) [2]. Apex cusp shape has traditionally been scored as sharp, round, or blunt, while relief (the difference in elevation between cusp and valley height) is scored as high or low [2]. Subsequent iterations of dental mesowear for horses adopted a scoring system from zero to three [5], zero to four [6], and zero to six [7], that encompassed high relief and sharp cusps (0) to blunt and low relief teeth (3, 4, or 6, respectively).

Blunted cusps and low relief across the occlusal surface are thought to be indicative of a phytolith-rich grazing diet (grasses also having a high-silica content and higher fibrousness that results in increased abrasion to the tooth surface during feeding) and/or one that includes a large amount of grit [2,8]; although grass is a more likely contributor than grit [8]. In contrast, browsers consume less siliceous foliage which causes less abrasion to an organism’s teeth; the occlusal wear on browse-dominant feeders allows for clean cuts and thus tooth-on-tooth attrition during mastication [2,8]. Mixed feeders may utilize both browse and grass materials and typically have intermediate mesowear characteristics (e.g., rounded cusps and medium relief) [2]. Most notably, mesowear has documented dietary evolution of equids in North America from browsers to a cosmopolitan group of mixed-feeders and grazers [7]. While the utility of dental mesowear has been expanded to include both upper and lower teeth [9] and a diversity of ungulates (e.g., antelopes, bovids, bison, camels, and deer) and even marsupials [5,8,10–16], some aspects of dental mesowear are not well understood (e.g., positive relationships between relief height and carbon isotope values [4,13], an unexpected relationship—one would predict reduced relief height with increased carbon isotope values indicative of C_4_ grass consumption).

Further, there are gaps in our knowledge of how dental mesowear relates to an organism’s local environment. As mesowear is largely documented to infer an animal’s diet, relationships with climate variables may be expected if diet varies with climate (through the lens of vegetation consumed; e.g., eating more grass in drier environments). That being said, increased dust and/or grit in drier environments may also lead to blunter and/or lower relief cusps in mammals inhabiting arid environments. While several studies have investigated the relationship between mesowear and climate (including precipitation and relative humidity [16,17]), results are equivocal. Mesowear may reflect local-to-regional relative humidity signals in wild African zebras with a high ratio of blunt and low relief cusps occurring in drier regions [17]. When a broad diversity of ungulates were examined, comparing average dental mesowear scores to precipitation at the center of a species range, no relationship was apparent [8]. Similarly, the mesowear of sika deer in Japan is not significantly correlated with precipitation [16]. Further investigation into the relationships between dental mesowear and climatic variables is warranted.

In Australia, marsupials are the dominant herbivores, including kangaroos, wallabies, wombats, possums, koalas, and others. To date, only one study of marsupial mesowear has been performed and demonstrated the potential of dental mesowear analysis for marsupial herbivores [15]. However, one of the most ubiquitous and spatially and temporally distributed kangaroos could not be appropriately classified (the eastern gray kangaroo, *Macropus giganteus*). Further, an additional 18 of 43 species could not be classified into appropriate dietary categories based on dental mesowear alone. It is unclear if and why dental mesowear is not highly effective in marsupials and/or if climate is heavily influencing dental mesowear variables.

**Fig 1:**
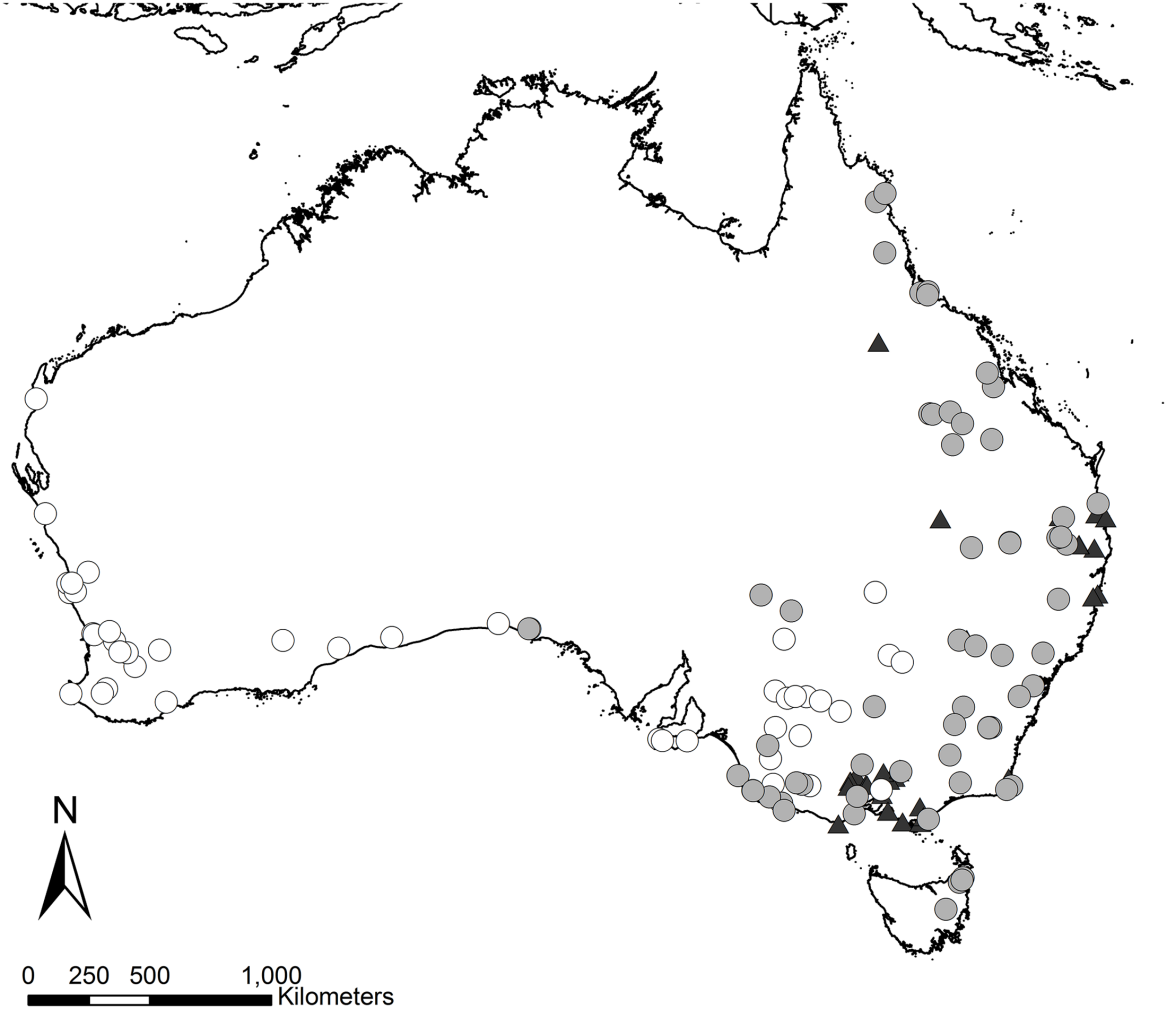
The location of *Macropus fuliginosus* (white circles), *Macropus giganteus* (gray circles), and *Phascolarctos cinereus* (black triangles) specimens used in this study.

Here, we examine the relationship between Australian marsupial mesowear, diet, and climate in three ubiquitous herbivores. We focus our analysis on extant species whose ranges are broad and dietary interpretations vary from grazing to mixed feeding: *Macropus giganteus* (a grazer to mixed feeder [15,18–21]), *Macropus fuliginosus* (a grazer to mixed feeder [15,20–22]). We also selected a species with a similarly broad range, which is an obligate browser and eucalyptus specialist, *Phascolarctos cinereus* [20,22,23]. We examined specimens from across Australia (including mainland Australia and Tasmania, when present; Fig 1, Tables A and B in S1 File) to ensure that taxa from a wide range of environments (that include a broad range of temperatures and evapotranspiration conditions, precipitation/relative humidity) would be sampled. We also examined mesowear across the cheek toothrow to understand how and/or if the inclusion of wear from multiple cusps affected dietary interpretations; however, unerupted/unworn molars were not included in this study. As conditions become more evaporative (i.e., higher temperature and/or lower precipitation/relative humidity), vegetation may become tougher and/or dust on vegetation may increase. Therefore, we test the ability of mesowear to recover such ecological signals through the following hypotheses:

1. Koalas and kangaroos can be distinguished from one another (as browsers and mixed feeders, respectively) via dental mesowear of mandibular teeth.
2. Specimens from more arid regions (lower annual precipitation and/or relative humidity) exhibit more abrasive mesowear (blunter and lower relief teeth) as compared to conspecifics from wetter regions.
3. Specimens from warm regions (higher mean minimum or maximum temperatures) exhibit less abrasive mesowear in koalas (due to increased soft-foliage consumption during a longer growing season), while kangaroos exhibit higher mesowear scores (blunter and lower relief teeth from increased grazing in warmer tropical environments with increased C_4_ grass presence) than conspecifics in cooler regions.

## Materials and methods

The specimens analyzed in this study consisted of 71 *Macropus fuliginosis* specimens, 96 *Macropus giganteus* specimens, and 46 *Phascolarctos cinereus* specimens from across Australia (Fig 1; Tables A-B in S1 File) from the Australian Museum (AM, Sydney, NSW, Australia), the Australia National Wildlife Collection (ANWC, Canberra, ACT, Australia), the Museum Victoria (MV, Melbourne, VIC, Australia), the Queensland Museum (QM, South Brisbane, QLD, Australia), the Western Australian Museum (WAM, Perth, WA, Australia), and collections at Flinders University (Adelaide, SA, Australia; individuals sampled for dental microwear by Ref. [24]).

**Fig 2:**
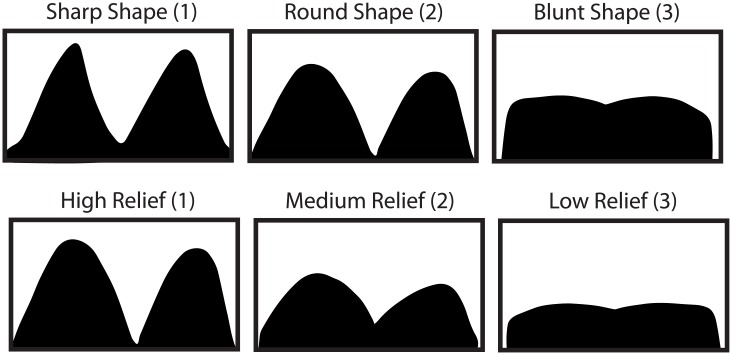
Examples of the mesowear variables used to score specimens and associated numeric values.

Each specimen with a mandible was analyzed from either a photograph (buccal profile) of the toothrow or an epoxy resin cast of the teeth. Mandibles were selected as mandibles and mandibular teeth are typically more common in Pleistocene localities in Australia than maxillas and maxillary teeth. We used the mesowear attributes most commonly used in the literature, scoring both shape and relief and a combined score (Fig 2). Cusp shape was scored as sharp (1), round (2), and blunt (3). Relief was scored on a slightly different scale from high (1), to medium (2), to low (3). While prior studies [2, 15] do not use a medium relief category, we found it helpful when intermediate relief was observed. Additionally, we used a combined score that totaled both scores and subtracted 2 from the resulting score ((Shape + Relief) - 2) = Combined Score), so as to result in a scale of 0–4 (much like Ref. [15], where 0 is equivalent to a tooth with sharp cusps and high relief while 4 is equal to a blunt tooth with low relief). We modified this score slightly from Ref. [15] (and others who have used scores ranging from 0 to 3/4/6) so that teeth with medium relief and sharp cusps and high relief teeth with round cusps were scored the same (with both conditions indicative of similar degrees of mesowear). In addition to scoring the sharpest cusp, per Ref. [2], we also scored all teeth in koalas (lower first through fourth molars, eight cusps on four teeth; see Tables C-D in S1 File) and two lower molars (four cusps) in kangaroos. In *M*. *giganteus* and *M*. *fuliginosus*, the two teeth (and subsequent four cusps) positioned to process the most vegetation (i.e., the teeth in occlusion and not erupting or in the process of being ejected due to molar progression [25]) were scored for mesowear analysis. Specimens with a lower m1 in occlusion and/or that had already been in occlusion (e.g., potentially worn and/or ejected) were examined; thus, the youngest individuals of all species were excluded. Each specimen was scored by six individuals consisting of undergraduate and graduate students; all had moderate to limited prior experience scoring mesowear cusps (note, Ref. [4] demonstrated the ability of novices to learn mesowear with a brief training session). Median scores were calculated for each specimen, minimizing the effect of the number of mesowear scorers while reducing mesowear observer variability (per Ref. [4], the results of mesowear analysis is improved by incorporating five or more observer scores per specimen).

Specimen metadata was gathered from the Atlas of Living Australia, a biodiversity database compiling information about occurrence records and specimens in natural history collections (Atlas of Living Australia [26]). Geographic occurrence data was converted to decimal degrees, if necessary. If geographic data was given via descriptive location data (e.g., 10 km due south of the town of Townsville), it was georeferenced to the finest resolution possible using Google Earth Pro [27].

Mean annual precipitation (MAP, mm) and mean maximum annual temperature (Max. MAT, °C) and mean minimum annual temperature (Min. MAT, °C) data were collected from the Australian Government Bureau of Meteorology weather station, closest to each location that possessed at least a decade of temperature and/or precipitation records (Australian Government Bureau of Meteorology [28]). The maximum distance between a specimen location and its corresponding weather station was less than 125 kilometers; the majority of specimens (87%) were within 50 km of both temperature and precipitation weather stations while 98% were within 100 km of both temperature and precipitation weather stations. When possible, thirty-year averages from 1961–1990 were used. These climate data have the potential to elucidate differences between regions and are more useful to characterizing an animal’s local environment than short term weather events. Relative humidity data were obtained from the NASA Langley Research Center Atmospheric Science Data Center Surface meteorological and Solar Energy (SSE) web portal supported by the United States National Aeronautics and Space Administration LaRC POWER Project [29]. In this dataset, relative humidity is measured at 10 m above the earth’s surface at a 1-degree resolution across the globe. The data are an average of values collected between July 1, 1983 to June 30, 2005 and downloaded on December 10, 2007. Relative humidity data was extracted from the NASA SSE layer using the spatial join tool available in ArcMap 10.4 [30].

Non-parametric Spearman’s rank-order correlation coefficients were calculated via XLSTAT [31] to assess if dental mesowear scores were correlated with the climate variables noted above. Further, the koalas and kangaroos were compared using non-parametric Kruskal-Wallis tests to assess if mesowear scores from mandibular teeth showed species differences.

## Results

### Diet and dental mesowear

**Fig 3:**
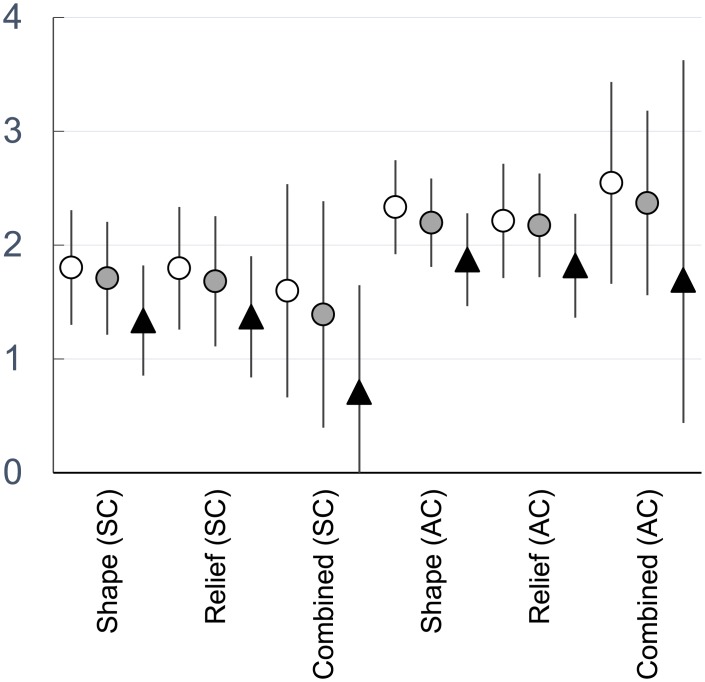
Comparison of dental mesowear scores (based on the sharpest cusp, SC, or average cusp scores, AC; see Materials and methods) amongst *M*. *fuliginosus* (white circles), *M*. *giganteus* (gray circle), and *P*. *cinereus* (black triangles). The bars represent the standard deviation of scores with the mean values noted by the presence of the respective symbols.

Descriptive statistics of dental mesowear scores are noted in Table 1 and summarized in Fig 3 (all primary data are noted in Tables A-D in S1 File). Dental mesowear from mandibular teeth of two species of extant kangaroos (*Macropus giganteus* and *Macropus fuliginosus*) have significantly lower relief (higher relief scores), lower combined scores (indicative of blunter and more worn teeth), and blunter shapes than koalas (*Phascolarctos cinereus*) via all mesowear scores analyzed (the sharper cusp and the combined analysis; p<0.01, Fig 3). *M*. *fuliginosus* has significantly blunter teeth (higher average cusp shape scores using the average of four cusps, see Materials and methods) than *M*. *giganteus* (p = 0.013) despite both being categorized as grazing/mixed-feeding; no other mesowear scores (relief or combined score) are significantly different between these taxa (p>0.05).

**Table 1:** Descriptive statistics of dental mesowear variables for the three target taxa here examined (*M*. *fuliginosus*, *M*. *giganteus*, and *P*. *cinereus*).

Species	Mesowear attribute	n	Min.	Max.	Range	Median	Mean	SD
*Macropus fuliginosus*	Sharpest cusp shape	71	1.0	3.0	2.0	2.0	1.803	0.503
	Sharpest cusp relief		1.0	3.0	2.0	2.0	1.796	0.538
	Sharpest cusp combined score		0.0	4.0	4.0	2.0	1.599	0.936
	Average cusp shape (4 cusps)		1.0	3.0	2.0	2.5	2.333	0.413
	Average cusp relief (4 cusps)		1.0	3.0	2.0	2.375	2.213	0.502
	Average cusp combined score (4 cusps)		0.0	4.0	4.0	2.875	2.546	0.886
*Macropus giganteus*	Sharpest cusp shape	96	1.0	3.0	2.0	2.0	1.708	0.496
	Sharpest cusp relief		1.0	3.0	2.0	2.0	1.682	0.572
	Sharpest cusp combined score		0.0	4.0	4.0	1.5	1.391	0.995
	Average cusp shape (4 cusps)		1.0	3.0	2.0	2.25	2.197	0.389
	Average cusp relief (4 cusps)		1.125	3.0	1.875	2.125	2.173	0.455
	Average cusp combined score (4 cusps)		0.625	4.0	3.375	2.375	2.370	0.811
*Phascolarctos cinereus*	Sharpest cusp shape	46	1.0	3.0	2.0	1.0	1.337	0.484
	Sharpest cusp relief		1.0	3.0	2.0	1.0	1.370	0.532
	Sharpest cusp combined score		0.0	3.0	3.0	0.0	0.707	0.940
	Average cusp shape (8 cusps)		1.25	3.0	1.75	1.844	1.872	0.407
	Average cusp relief (8 cusps)		1.188	3.000	1.813	1.750	1.819	0.456
	Average cusp combined score (8 cusps)		0.438	3.625	3.188	1.531	1.692	0.818

n, number of specimens; Min., minimum; Max., maximum; Range, total range; SD, standard deviation (n-1).

### Climate and dental mesowear

**Fig 4:**
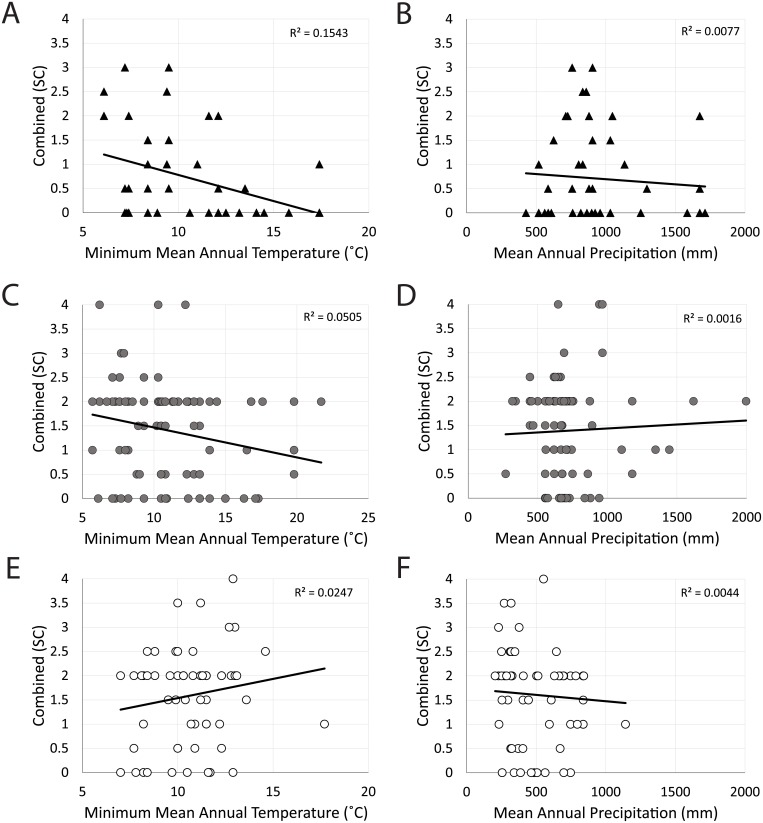
Bivariate plots denoting relationships between climate variables (Min. MAT, °C; MAP, mm) and the sharpest cusp combined score amongst *P*. *cinereus* (black triangles; A, B), *M*. *giganteus* (gray circles; C, D), and *M*. *fuliginosus* (white circles; E, F).

Relationships between dental mesowear and climate variables (including correlation coefficients and p-values) are noted in Fig 4 and Table 2 (all primary data, including information regarding climate stations and corresponding metadata, are noted in Tables A-D in S1 File). None of these species exhibit significant correlations between any mesowear attribute and precipitation (MAP) or relative humidity (all p-values>0.1). For koalas, Min. MAT is negatively correlated with the relief and combined scores (both the traditional sharpest cusp method and an alternate method of using the average shape of multiple cusps (4 in kangaroos and 8 in koalas; p<0.02; Table 2; Tables A-D in S1 File). Note that for koalas, there are no significant relationships between either mesowear shape score and Min. MAT. Additionally, koala relief scores are negatively correlated with Max. MAT, such that greater Max. MAT results in higher relief (all p<0.04; Table 2).

**Table 2:** Summary of Spearman correlation coefficient (rho) values for each dental mesowear attribute examined for each species. All bold values indicate significance (p<0.05).

Species	Mesowear attribute	Lat.	Long.	MAP	RH	Min. MAT	Max. MAT
*Macropus fuliginosus*	Sharpest cusp shape	-0.07	0.03	-0.09	-0.04	0.11	0.05
	Sharpest cusp relief	-0.11	-0.07	-0.06	0.01	0.14	0.01
	Sharpest cusp combined score	-0.10	0.00	-0.11	-0.03	0.10	0.03
	Average cusp shape (4 cusps)	-0.15	0.01	-0.19	-0.09	0.14	0.11
	Average cusp relief (4 cusps)	-0.15	-0.02	-0.11	-0.03	0.17	0.02
	Average cusp combined score (4 cusps)	-0.17	0.03	-0.15	-0.08	0.17	0.07
*Macropus giganteus*	Sharpest cusp shape	**0.21**	**-0.20**	-0.04	0.07	**-0.22**	-0.19
	Sharpest cusp relief	0.18	**-0.22**	-0.06	0.01	**-0.25**	-0.16
	Sharpest cusp combined score	**0.21**	**-0.26**	-0.06	0.03	**-0.27**	-0.20
	Average cusp shape (4 cusps)	**0.28**	-0.19	-0.06	0.10	**-0.25**	**-0.23**
	Average cusp relief (4 cusps)	**0.25**	**-0.26**	-0.05	0.06	**-0.29**	-0.20
	Average cusp combined score (4 cusps)	**0.27**	**-0.23**	-0.06	0.08	**-0.27**	**-0.21**
*Phascolarctos cinereus*	Sharpest cusp shape	0.15	0.08	0.03	0.23	-0.24	-0.08
	Sharpest cusp relief	**0.39**	-0.12	-0.03	0.1	**-0.50**	**-0.32**
	Sharpest cusp combined score	0.27	-0.03	0.0	0.17	**-0.39**	-0.19
	Average cusp shape (8 cusps)	0.20	-0.03	-0.01	0.19	-0.24	-0.14
	Average cusp relief (8 cusps)	**0.45**	-0.13	0.08	0.18	**-0.43**	**-0.37**
	Average cusp combined score (8 cusps)	**0.33**	-0.09	0.04	0.19	**-0.35**	-0.26

Lat., latitude (absolute value of degrees South); Long., Longitude (degrees East); MAP, mean annual precipitation (mm); RH, relative humidity (%, as defined in Materials and methods); Min. MAT, mean minimum annual temperature (°C); Max. MAT, mean maximum annual temperature (°C).

*Macropus giganteus* dental mesowear scores (all six variables) are negatively correlated with Min. MAT (all p<0.04; Table 2), similar to koalas. Further, dental mesowear average cusp shape and the average cusp combined score is negatively correlated with Max. MAT, such that teeth are sharper in regions with higher average maximum temperatures (all p<0.04). Additionally, no significant relationships were found between any mesowear scoring methods and any of the climate variables here examined (i.e., relative humidity, precipitation, or annual minimum or maximum temperature) in *Macropus fuliginosus* (p>0.05), despite the specimens examined occurring in states exhibiting significantly different climate variables (based on non-parametric Kruskal-Wallis comparisons of climate variables from specimens of *M*. *fuliginosus* in different states, p≤0.0001; however, the variability in mean annual temperature is less than in the other two taxa). Some relationships between climate variables and *M*. *giganteus* mesowear are stronger using the average of multiple cusps while those in koalas show mixed results (some are slightly weaker while others are slightly stronger, using the average of multiple cusps; Table 1).

## Discussion

Dental mesowear is a valuable method for assessing mammalian diets due to its simplicity and ease of use [2,4]; however, it is often unclear if and how climatic factors (and subsequent dust/grit loads) affect dental mesowear [16,17]. Based on the dental mesowear of the mandibular teeth of three ubiquitous taxa with broad geographic ranges that traverse different climatic regimes, one being an obligate browser and the other two eating a mixture of grass and browse, dental mesowear does capture dietary differences. Our first hypothesis, that there is a statistically significant difference between the mandibular mesowear of kangaroos and koalas with disparate diets, is accepted (Fig 3). Our second hypothesis, that specimens from more arid regions exhibited more wear, was rejected; there are no statistically significant relationships between precipitation and/or relative humidity and any of the mesowear scoring methods for any of the three species. For our third hypothesis, koalas living in cooler environments (as defined by lower mean minimum temperatures) had more abrasive wear—as hypothesized. However, *Macropus giganteus* exhibited the same relationship as koalas, contrary to expectations that *M*. *giganteus* consumes more abrasive grasses in warmer regions. *Macropus fuliginosus* had no statistically significant correlations to any of the climate variables, despite significant differences in climate variables for the subset of *M*. *fuliginosus* specimens examined; however, the total temperature range is less than that of the other two taxa.

Previous work demonstrated the potential of dental mesowear in marsupial herbivores [15]; however, one of the most ubiquitous kangaroos could not be classified as mixed-feeders based on dental mesowear and were excluded from subsequent analyses with extinct taxa (*M*. *giganteus*). While we do document significant differences between these grazing/mixed-feeding kangaroos and browsing koalas, our data set does not include the broad array of herbivorous marsupials included in the prior study [15]. Further, these differences appear less pronounced as between ungulate taxa which may also be related to the lower time window during which dental mesowear can form on taxa exhibiting molar progression. While Ref. [15] included dozens of species (24–43 species), the sample size of each species was often restricted to specimens from Queensland and/or other states with a state focused collection (and limited to ~10–30 specimens). Here, we trade the diversity of specimens for a more in-depth analysis of fewer ubiquitous taxa, each distributed over a broad range of latitudes and/or longitudes. Fundamentally, diet appears to be recorded via dental mesowear in maxillary teeth, in a subset of their data [15]; while we here demonstrate that dental mesowear is recorded in mandibular teeth in a subset of taxa. Future work should compare maxillary to mandibular teeth. Further, as we here demonstrate, dental mesowear can vary across regions and may demonstrate subtle differences in diet across a species range (although we do not rule out ontogenetic differences, adults were primarily analyzed). Thus, dental mesowear can further be touted as an easy and accessible dietary proxy useful for reconstructing ancient diets of herbivores through time and in response to climate change. With the expansion of digital natural history museum archives, including photographs of both modern and fossil specimens, the use of dental mesowear will likely continue to help clarify diets in a broad range of taxa, including adding marsupial herbivores to the list of focal taxa.

None of the mesowear scoring methods for any of the species we examined showed a significant positive or negative relationship with precipitation and/or relative humidity. This is contrary to expectations that regions with lower mean annual precipitation and/or lower relative humidity would have increased grit and/or dust on vegetation resulting in more worn teeth (i.e., lower relief and combined scores)—if grit/dust and not diet are primarily contributing to dental mesowear formation. Instead, there are greater differences in dental mesowear between taxa with different diets than there are between conspecifics occurring in regions experiencing different climatic regimes. These data confirm patterns observed in prior studies [8,16, 32], most notably that diet is the primary signal recorded via dental mesowear (i.e., it is grass not grit that is likely being recorded via dental mesowear). Of the climatic variables analyzed, only variations in temperature were shown to affect mesowear. This may be due to changes in dietary resources in *Macropus giganteus* and *Phascolarctos cinereus* in regions experiencing different temperatures.

Koala mandibular teeth from warmer regions showed lower mesowear values (higher relief and lower combined scores), consistent with our third hypothesis that mesowear scores in koala mandibular teeth will decrease with increased temperature. As suggested by Ref. [33], this may be due in part to the need for thermoregulation. Koalas exhibit increased panting when temperatures increase to facilitate evaporative cooling, leading to increased water loss [33]. As such, plants with higher water content are preferred in order to regulate body temperature and restore water balance [33]. Assuming leaves with higher water content are also less abrasive, this dietary strategy may lead to reduced wear (and subsequently higher relief and less blunting of the tooth cusps). Similarly, it has also been suggested that when vegetation is abundant, koalas exhibit a preference for new growth [34–36], presumably because these leaves are softer, easier to chew, and contain a higher water and/or nutrient content (with dental microwear of koalas also demonstrating the consumption of less abrasive leaves in drier regions [37]. Koalas in cooler regions where growing seasons are shorter, and body sizes and fur depth are larger and deeper, respectively [38], consume more vegetation due to higher metabolic demands (much like has been documented seasonally, with koalas eating more in the winter than the summer [39]), resulting in increased gross wear—including lower relief (higher values), documented here.

Much like koalas but contrary to predictions, *Macropus giganteus* showed significant negative correlations of dental mesowear scores with temperature. This is contrary to what we predicted. Similar to our initial predictions that drier regions contribute to blunter teeth (potentially due to dust/grit), we also expected *M*. *giganteus*, which is known to consume predominantly C_4_ grasses in warmer and more tropical (and lower latitude) regions [24] (and per communication with G. Prideaux, the specimens here included from Flinders University consumed primarily C_4_ grass based on stomach content analysis and/or stable carbon isotope analysis) to have teeth with lower relief, blunter shapes, and higher combined scores in these regions, in contrast to higher/cooler latitudes. While classified as grazers (≥70% grass) [21] and mixed feeders (<90% grass) [15] they are both known to vary their diet throughout their geographic range [40]; thus, dietary behavior may be more complex, challenging the use of simplified dietary categories. Further, while both Refs. [15,21] review the literature and provide consensus views of dietary interpretations, Ref. [41] estimated percent grass consumption based on stable carbon isotopes of bone collagen and tooth enamel in kangaroos. Specifically, they estimated that *M*. *fuliginosus* consumed less than 40% grasses, while *M*. *giganteus* consumed nearly 100% grasses, both based on bone collagen (with enamel estimates of *M*. *fuliginosus* suggesting increased grass consumption, yet still less than 60%) [41]. Other studies document highly variable estimates for % grass consumption in *M*. giganteus, ranging from ~50% [19] to between 64–84% [18]. Further, studies of habitat selection in areas where these two species co-occur, suggest that *M*. *giganteus* selects habitats with a larger proportion of grasses and lower mean lateral cover (%), as compared to *M*. *fuliginosus* [42]. Thus, it is rather surprising that *M*. *giganteus* had teeth with sharper cusps and higher relief in areas with a higher incidence of C_4_ grass. Further work is needed to better understand how grass consumption affects dental mesowear in kangaroos exhibiting molar progression, building off of the work of Ref. [44]. Specifically, molar progression and rates of tooth wear are decoupled, with gross tooth wear varying in both *M*. *fuliginosus* and *M*. *giganteus* between individuals of the same population at the same time, between individuals within the same region at different times, and between different populations from different regions [44]; yet, little is understood regarding how this affects dental mesowear. Additional work is also needed to assess if the incidence of hard quartz grains varies with latitude along eastern Australia, which may have also affected koalas and kangaroos similarly.

Several limitations should be noted in this study. Climate data for each specimen was dependent on distances between the location of specimens and weather stations, some of which included distances of ~100 km (although, this only applied to a subset of specimens, see Materials and methods). Further, while collection data including the year a specimen was collected is often available for museum specimens, many times climate data corresponding with those years is not available (and/or it is unclear under what duration of time the specimen was alive and how many years or months of data should be included with that specimen). For these reasons, we generally characterized a regions climate using 1961–1990 year averages. Further, many of these taxa can have broad home ranges during the life of the individual specimen [43], and unlike dental microwear which captures the "last supper" (past few days to weeks of an animal’s diet [45]), dental mesowear is cumulative—capturing a multi-season and multi-year dietary average [2,6]. Experimental studies examining modern populations of kangaroos and/or studies of kangaroos from specific regions that experienced non-drought and pronounced drought conditions could improve our understanding of how diet may vary with climate change, and/or if grit does substantially impact mesowear. Further work is also needed to evaluate if ontogenetic differences influence diet, as inferred from dental mesowear (i.e., molar progression; if mesowear is most telling on teeth with the longest ‘lifespan’).

Despite early work which suggests that mesowear may be a function of both diet and grit on the landscape [2], it appears that diet is the overarching signal recorded in both individual teeth and multiple teeth across the tooth row—at least for the marsupials discussed here. This is consistent with prior work documenting a significant positive relationship between percent grass in an animal’s diet and mesowear score (0–4) while no relationships existed between mean annual precipitation and mesowear scores [8]. While more work is needed to better determine the efficacy and/or benefits of scoring multiple teeth (instead of or in addition to the sharpest cusp), average shape scores across the entire tooth row do differentiate these two mixed feeding kangaroos from one another, which may be eating slightly different foods (either on average or in different regions) from one another—showing that nuanced differences can be garnered in certain cases using the cumulative method. In addition to gaining valuable insights into mesowear variability in different species, we document the absence of any relationship between dental mesowear attributes and aridity (either precipitation or relative humidity). Further, relationships between dental mesowear and temperature are likely related to dietary differences across regions, although further work is needed to experimentally test these relationships.

## Supporting information

S1 File Supplementary Tables A-D noting dental mesowear attribute values and associated climate and metadata per specimen examined for all taxa analyzed here, including: *Macropus giganteus*, *Macropus fuliginosus*, and *Phascolarctos cinereus*. (XLSX)
